# Critical comparative analysis of data sources toward understanding referral during pregnancy and childbirth: three perspectives from Nigeria

**DOI:** 10.1186/s12913-021-06945-9

**Published:** 2021-09-06

**Authors:** Emma Radovich, Aduragbemi Banke-Thomas, Oona M. R. Campbell, Michael Ezeanochie, Uchenna Gwacham-Anisiobi, Adedapo B. A. Ande, Lenka Benova

**Affiliations:** 1 Faculty of Epidemiology and Population Health, London School of Hygiene and Tropical Medicine, London, UK; 2 LSE Health, London School of Economics and Political Science, London, UK; 3 Department of Obstetrics and Gynaecology, University of Benin Teaching Hospital, Benin, Edo State Nigeria; 4 Health Strategy and Delivery Foundation, Owerri, Imo State Nigeria; 5 Department of Public Health, Institute of Tropical Medicine, Antwerpen, Belgium

**Keywords:** Maternal health, Newborn health, Emergency obstetric care, Referral, Health systems, Facility-based birth, Data, Medical records, Household survey, Nigeria

## Abstract

**Background:**

The highest risk of maternal and perinatal deaths occurs during and shortly after childbirth and is preventable if functional referral systems enable women to reach appropriate health services when obstetric complications occur. Rising numbers of deliveries in health facilities, including in high mortality settings like Nigeria, require formalised coordination across the health system to ensure that women and newborns get to the right level of care, at the right time. This study describes and critically assesses the extent to which referral and its components can be captured using three different data sources from Nigeria, examining issues of data quality, validity, and usefulness for improving and monitoring obstetric care systems.

**Methods:**

The study included three data sources on referral for childbirth care in Nigeria: a nationally representative household survey, patient records from multiple facilities in a state, and patient records from the apex referral facility in a city. We conducted descriptive analyses of the extent to which referral status and components were captured across the three sources. We also iteratively developed a visual conceptual framework to guide our critical comparative analysis.

**Results:**

We found large differences in the proportion of women referred, and this reflected the different denominators and timings of the referral in each data source. Between 16 and 34% of referrals in the three sources originated in government hospitals, and lateral referrals (origin and destination facility of the same level) were observed in all three data sources. We found large gaps in the coverage of key components of referral as well as data gaps where this information was not routinely captured in facility-based sources.

**Conclusions:**

Our analyses illustrated different perspectives from the national- to facility-level in the capture of the extent and components of obstetric referral. By triangulating across multiple data sources, we revealed the strengths and gaps within each approach in building a more complete picture of obstetric referral. We see our visual framework as assisting further research efforts to ensure all referral pathways are captured in order to better monitor and improve referral systems for women and newborns.

**Supplementary Information:**

The online version contains supplementary material available at 10.1186/s12913-021-06945-9.

## Background

Despite the 38% reduction in global maternal deaths between 2000 and 2017 [1], over two-thirds of global maternal deaths occurred in sub-Saharan Africa in 2017 (196,000), with Nigeria alone accounting for 23% of these global maternal deaths [1]. Globally, the highest risk of maternal and perinatal death is around the time of childbirth, with direct obstetric causes (such as severe bleeding, hypertensive disorders, and sepsis) which require urgent intervention, accounting for more than half of maternal deaths [1, 2]. Timely access to skilled health personnel and health facilities capable of providing the requisite care is critical for reducing negative pregnancy outcomes among women and their newborns [3–5]. Evidence shows that maternal and perinatal deaths can be prevented if women are at the right level of care to manage complications or if functional referral systems are in place to enable women to reach appropriate health services on time when obstetric complications occur [6].

Much of the literature around access to care during obstetric emergencies has been framed around Thaddeus and Maine’s seminal three-delays framework [7]. The first delay is in deciding to seek care, the second delay in identifying and reaching a medical facility, and the third delay is in receiving adequate care at the facility. The three-delays framework arose when the vast majority of deliveries in low- and middle-income countries (LMICs) took place in home environments. However, the landscape of childbirth care in LMICs has shifted with a significant rise in the proportion of births in health facilities [8]. Some women bypass the lowest level of care (primary) and directly seek care in higher-level (secondary+) health facilities which should have the capability to manage common complications, although many women in high-mortality settings still give birth in facilities incapable of providing this higher-level care, referred to as comprehensive emergency obstetric care (CEmOC) [9–11]. Weak referral systems with little coordination between levels of care, as well as between the public and private sectors, mean that women and their families must frequently navigate the referral process on their own, including arranging their own transport, travelling through often bad road networks, at substantial financial costs [12–15]. In many sub-Saharan African countries, emergency obstetric referrals are delayed, involve multiple facilities before reaching appropriate care, including back and forth transfer between facilities with similar capabilities, as well as little communication and follow-up between referring and receiving facilities [13, 16].

An effective referral system involves formalised coordination across the health system, including transportation and communication, to ensure women and newborns receive timely access to specialist care in the event of complications [10]. The 2016 Lancet Maternal Health Series highlighted how little is known about the design, use and monitoring of optimal referral pathways to more advanced care [17]. Previous efforts to monitor obstetric referral in LMICs have used aggregated health management information systems (HMIS) or facility survey data [18, 19], and qualitative studies have detailed women’s and families’ experiences of referral [13, 16]. However, few studies have documented what happens during obstetric referral and the outcomes for women and newborns at large scale. A key gap in the literature exists to critically assess how different obstetric referral data sources can address important questions such as: Who is being referred and why? Where are they coming from and going to? How are they travelling and who is travelling with them? And finally, what are the outcomes of women who are referred and their babies? The Coronavirus Disease (COVID-19) pandemic, during which models of care delivery and continuity have been disrupted and adapted to fit the prevailing circumstances, has further exposed the need for a coordinated response across levels of the health system to ensure women and newborns receive appropriate and timely care during complications [10, 20].

The objective of our study is to describe and critically assess the extent to which obstetric referral status and its components can be captured in various existing data collection systems. To construct this case study, we used three independent data sources from Nigeria: a nationally representative household survey, patient records from multiple facilities in a State (sub-national level), and patient records from the apex referral facility in a city (facility level). We report results and discuss issues of data quality, validity, and usefulness to improve and monitor systems of obstetric care provision.

## Methods

### Framework

To guide our critical comparative analysis, we iteratively developed a visual conceptual overview, using principles defined by Miles and Huberman [21]. As data inputs, we used our technical knowledge and research experience, a rapid literature review focussing on obstetric referral, and other conceptual approaches and visuals depicting the pathways and journeys of women referred during labour and childbirth [13, 22–25]. We also engaged with the broader literature on maternal health service utilisation, including issues related to health-seeking behaviour [26, 27], bypassing [28–31], socio-economic determinants of health [32–34], physical availability of care [35, 36], detention of women in facilities due to inability to pay [37, 38], and broader health system design frameworks for maternal and newborn health [10, 17, 39–41]. Last, we were informed by issues and questions raised by the intersection of the three datasets used in this analysis.

**Fig. 1:**
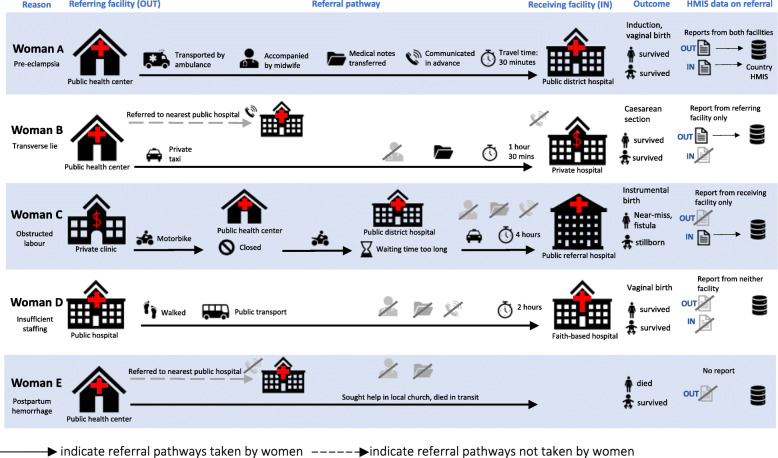
Conceptual framework of obstetric referral pathways

In the resulting conceptual framework (Fig. 1), we illustrate the various elements of referral and opportunities for capturing data on the process of referral, by showing hypothetical referral pathways of five women. We highlight that in addition to direct referral pathways along the hierarchy of the health system (Woman A), there are several complexities of referral pathways. These include bypassing of facilities during referral (Woman B), “zigzagging” between facilities (Woman C), lateral referrals between facilities of the same level or CEmOC capacity (Woman D), and incomplete referrals, resulting in the woman’s return to the facility of origin, to her home, or her death (Woman E). Within this visual, we also schematically highlight the various aspects of referral, including the reasons for referral, the type (level and sector/ownership) of health facilities involved, time and means of transport, whether the woman was accompanied by a skilled health personnel, whether clinical records and information about the referral were communicated between the referring (origin) facility and the receiving (destination) facility, maternal and perinatal outcomes, and potential sources of data about the referral.

### Description of setting and data sources

In Nigeria, formal childbirth care is arranged hierarchically; tertiary healthcare, often provided by University Teaching Hospitals, is at the apex of the system and manages the most difficult cases that are referred from the lower tiers of the health system. Recommendations for managing obstetric complications from the Society of Gynaecology & Obstetrics of Nigeria state that providers should “refer the patient to a higher level of care at any stage if there are challenges with the drugs or delivery” and recommend that the referred patient is accompanied by a healthcare provider and with relevant documentation [42, 43]. However, a systematic review by Hussein and colleagues identified ineffective referral as one of the four main barriers to receiving life-saving obstetric care in Nigeria [12].

This study includes three data sources (Table 1) on referral for childbirth care: 1) the national Nigeria Demographic and Health Survey (NDHS) 2018, 2) state-level facility patient case notes from all government CEmOC facilities in Lagos state, and 3) patient case notes from University of Benin Teaching Hospital (UBTH) in Benin City, Edo State.

**Table 1:** Summary of three data sources

	Data source 1 NDHS 2018	Data source 2 Government CEmOC facilities, Lagos state	Data source 3 UBTH, Benin City, Edo State
**Study site**	National sample of households in Nigeria	All public CEmOC hospitals in Lagos State	Apex referral hospital in Benin City, Edo State
**Source of information**	Woman’s self-report, recalled up to 5 years before survey	Patient hospital records	Patient hospital records
**Study type**	Cross-sectional household survey	Retrospective census of patient records	Retrospective census of patient records
**Data collection period**	14 August 2018–29 December 2018	14 September 2019–2 March 2020	7 August 2018–21 August 2018
**Time of births included in study**	1 January 2013–29 December 2018	1 November 2018–31 December 2019	1 December 2017–31 July 2018
**Study population**	Women with a live birth	Pregnant women who presented in emergency at maternity wards in one of the 24 government (federal and state)-owned facilities (conception to 7 days postpartum)	‘Un-booked’ pregnant women admitted to the UBTH maternity ward in the perinatal period (23+ weeks gestation to 7 days postpartum)
**Perinatal outcome measures used in analysis**	Birth by caesarean section, Early neonatal death (within 7 days after delivery)	Birth by caesarean section, Maternal death, Stillbirth, Early neonatal death	Birth by caesarean section, Maternal death, Stillbirth

Data source 1 was the 2018 NDHS, a cross-sectional, nationally representative household survey using a multi-stage cluster sampling design. The NDHS used the standard model questionnaires including ﻿the Woman’s Questionnaire [44], which was adapted for country priorities. It also included a new set of questions on obstetric referral (Additional file 1), developed by the co-authors (ER, OMRC, LB) in collaboration with the Nigerian Federal Ministry of Health and implementing agencies. Respondents were women of reproductive age (15–49 years) at the time of the survey, who reported on the circumstances of their live births occurring in a five-year recall period. The unit of analysis was a live birth (multiple births per woman possible).

Data source 2 was a study conducted in Lagos State; data were collected from all 24 government-owned CEmOC facilities, which along with linked primary health care facilities account for two-fifths of all births in Lagos State [45]. Based on Lagos State Ministry of Health data, the facilities conducted between 120 (Apapa General Hospital) and 3681 (Lagos Island Maternity Hospital) deliveries in the year 2018. To capture data, one of the co-authors (ABT) with a team of medical doctors working in Lagos public hospitals reviewed case notes of all pregnant women who presented with obstetric complications at emergency maternity wards of these facilities during the year preceding the survey, including those recorded as ‘booked’ and ‘un-booked’.

Data source 3 was a study conducted in UBTH, the top referral facility for maternity services in Edo State. The facility performs between 3000 and 4000 deliveries annually. We conducted a retrospective review of the medical records of ‘un-booked’ women who were admitted in the perinatal period to the UBTH maternity ward between 1 December 2017 and 31 July 2018. ‘Un-booked’ denotes women who had not received antenatal care at UBTH and were therefore not registered or expected to give birth there. The free-form, handwritten case notes were reviewed by UBTH obstetric residents to extract the relevant data. See Additional file 2 for further study details.

### Data collection and definitions

Depending on availability, data were collected on demographic characteristics (including age, marital status, employment and education), obstetric history (parity, gestation, and mode of delivery), clinical management, status and origin of referral, support during referral, mode of travel to reach the receiving facility and pregnancy outcomes for women and newborns.

Across the three sources, we categorised women’s age as < 20 years, 20–34 years and ≥ 35 years. For data sources 2 and 3, women were classified as ‘booked’ if they were registered for antenatal care or delivery in the facility where they received CEmOC. For all data sources, we categorised facility sector as either government (public) or private (includes all for-profit, faith-based, and non-governmental facilities) and additionally distinguished, where possible, by the type or level of facility: hospital, primary or lower level, and others. Where available, we also captured data on women who came from non-facility locations, such as traditional birth attendant (TBA) homes or religious settings (such as churches and mosques).

We classified births as having been referred based on whether women reported that they “came from another health facility” prior to coming to the facility where they delivered (data source 1) or if the medical case notes indicated that the woman had been referred (data sources 2 and 3). In order to assess perinatal outcomes, we used the World Health Organization (WHO)’s definition of the perinatal period to capture > 22 weeks’ gestation and up to 7 days following childbirth. For data source 2, we included women across the entire pregnancy spectrum (conception to 7 days postpartum), but for data source 3, we limited the inclusion criteria to only pregnancies of 23+ completed weeks gestation to reduce potential misclassification of stillbirths and miscarriages due to inaccurate gestational age estimation (Table 1) [46]. We classified mode of delivery as vaginal, caesarean, or evacuation before viability (induced or spontaneous abortion).

### Analysis

We conducted descriptive analyses of the extent to which referral status and components were captured across the three sources. We estimated the proportion of the sample that was referred based on available and feasible denominators. Demographic, pregnancy and referral pathway data were summarised for referred women and presented in tables. Depending on data availability, we summarised and presented data on the day/period of the day the journey to the facility commenced, origin (or referring) facility type, the means of transportation used to reach the facility for delivery, number of referrals made, stop-overs made en route, whether a healthcare worker accompanied the woman from the origin facility to the facility where she gave birth, and whether the woman had a referral note. We presented the extent of missingness for all variables. For data source 1, we used the Sankey pathway to map referral pathways of women across different facility types.

Chi-squared tests were performed to compare proportions giving birth by caesarean as well as maternal and newborn outcomes (maternal death and stillbirth, respectively) between referred and non-referred women. STATA version SE 15.0 (StataCorp, College Station, Texas, USA) was used for data analysis.

We then conducted a critical assessment of the three data sources based on the components of the framework (Fig. 1). We reflected on issues of comparability and the strengths and gaps of the three data sources using a traffic light colour system for ease of display.

## Results

### Data source 1: NDHS 2018 – national perspective

Among all 34,193 live births in the survey’s five-year recall period, 13,462 (39.4%) were in health facilities. There was a wide variation especially by geographic zone and household wealth (Table 2). Among all live births, 13.1% were in government hospitals, and this was also socio-economically patterned. Nationally, 208 (1.5, 95%CI: 1.3–1.8) of live births in health facilities were reported to have been referred from another health facility. No statistically significant difference in the percentage of referrals was observed by geographic region, urban/rural residence, maternal education, or household wealth. Among births in government hospitals, 2.3% came from referrals; this differed across the regions from 1.0% (South East) to 3.9% (North East).

**Table 2:** Description of live births in the five-years before the survey, according to location of birth and referral status, NDHS 2018

	Location of births						Referrals					
	% of all births in health facilities		% of all births in government hospitals		% of all facility births which are in government hospitals		% of births in health facilities which came from referrals		% of births in government hospitals which came from referrals		% of all referrals which resulted in a birth in a government hospital	
*n*	*34,193*		*34,193*		*13,462*		*13,462*		*4490*		*208*	
	%	95%CI	%	95%CI	%	95%CI	%	95%CI	%	95%CI	%	95%CI
Overall	39.4%	37.8–41.0	13.1%	12.3–14.0	33.4%	31.7–35.0	1.5%	1.3–1.8	2.3%	1.8–3.0	50.4%	41.6–59.1
Region												
North Central	**49.2%**	45.9–52.5	**21.2%**	19.2–23.3	**43.0%**	40.0–46.2	0.9%	0.6–1.5	**1.3%**	0.7–2.5	**59.4%**	44.1–73.1
North East	**25.4%**	22.5–28.5	**10.5%**	8.6–12.6	**41.2%**	35.6–47.0	1.9%	1.2–2.9	**3.9%**	2.3–6.4	**85.0%**	64.7–94.6
North West	**15.6%**	13.6–17.7	**9.6%**	8.2–11.2	**61.5%**	56.8–66.0	2.2%	1.5–3.2	**2.0%**	1.2–3.4	**57.3%**	35.1–77.0
South East	**81.8%**	78.5–84.7	**10.2%**	8.5–12.1	**12.5%**	10.5–14.8	1.2%	0.8–1.7	**1.0%**	0.4–2.8	**10.8%**	3.6–28.1
South South	**50.2%**	46.2–54.2	**15.7%**	13.3–18.5	**31.3%**	26.8–36.2	1.8%	1.0–3.2	**2.5%**	1.3–5.1	**44.5%**	19.8–72.3
South West	**76.3%**	73.2–79.2	**19.2%**	17.2–21.4	**25.1%**	22.8–27.6	1.7%	1.2–2.3	**3.2%**	2.0–5.1	**49.1%**	21.3–67.0
Residence												
Urban	**61.1%**	58.4–63.6	**20.7%**	19.1–22.3	33.8%	31.7–36.1	1.6%	1.3–2.0	2.0%	1.5–2.8	**43.4%**	32.3–55.2
Rural	**25.8%**	24.2–27.5	**8.4%**	7.6–9.3	32.7%	30.2–35.3	1.5%	1.1–1.9	2.8%	2.0–3.9	**61.5%**	48.5–73.1
Maternal education												
No education	**13.8%**	12.7–15.1	**5.8%**	5.1–6.5	**41.6%**	38.4–44.9	2.0%	1.3–2.9	2.9%	1.7–4.7	60.6%	40.7–77.6
Primary	**40.5%**	38.3–42.8	**13.6%**	12.0–15.3	**33.5%**	30.0–37.2	1.3%	0.9–2.0	2.0%	1.1–3.7	51.3%	30.9–71.4
Secondary	**64.6%**	62.7–66.5	**18.6%**	17.3–20.1	**28.2%**	26.8–30.9	1.4%	1.1–1.7	2.3%	1.6–3.3	48.7%	36.0–61.5
Higher	**87.7%**	85.6–89.5	**33.5%**	31.0–36.2	**38.2%**	35.3–41.2	1.9%	1.3–2.8	2.1%	1.3–3.4	43.6%	25.5–63.5
Household wealth quintile												
Poorest	**11.6%**	10.2–13.1	**3.7%**	3.0–4.4	31.6%	27.5–36.0	1.7%	0.8–3.6	2.5%	0.6–10.3	46.2%	13.3–82.8
Poor	**21.1%**	19.1–23.2	**6.7%**	5.7–7.9	31.9%	28.1–35.9	1.8%	1.2–2.7	4.1%	2.5–6.7	74.2%	53.1–87.9
Middle	**40.3%**	37.6–43.0	**14.0%**	12.7–15.5	34.8%	31.7–38.1	1.6%	1.1–2.1	2.4%	1.5–3.8	53.5%	37.1–69.2
Richer	**59.2%**	56.7–61.6	**20.0%**	18.3–21.8	33.8%	30.9–36.8	1.7%	1.2–2.3	1.9%	1.2–2.9	38.9%	24.1–56.1
Richest	**79.5%**	77.0–81.8	**26.2%**	24.2–28.4	33.0%	30.4–35.6	1.3%	1.0–1.8	2.0%	1.3–3.1	49.2%	33.3–65.3

*Variables shown in bold denote chi-square test *p* < 0.05

Among referred cases (n = 208), the facility of origin was majority government lower-level facilities (58.4%); 19.7% came from private facilities, and 21.9% from government hospitals (Table 3). While half of referred cases gave birth in government hospitals (50.4%), 9.4% gave birth in government lower-level, and 40.2% in private facilities. The distribution of referred cases across levels of destination facility showed a large geographic variation. No differences in the distribution of referred cases by type of facility were observed by women’s residence, education, or household wealth. The most common transport used during referral was a private car/taxi (62.8%) or another form of motorised transport (30.2%). Ambulance was reported to have been used by 3.4% of the referred cases. Only a quarter of women said they were accompanied by a health professional during the referral.

**Table 3:** Characterisation of cases referred during the intrapartum period, NDHS 2018

	**Among referred cases**				
	*n = 208*				
	%	95%CI			
**Facility of origin**					
Government hospital	21.9	15.8–29.5			
Government lower-level facility	58.4	50.6–65.9			
All private sector	19.7	14.0–27.0			
**Facility type where woman gave birth**					
Government hospital	50.4	41.6–59.1			
Government lower-level facility	9.4	6.1–14.4			
All private sector	40.2	31.9–49.1			
**Means of transport**					
Ambulance	3.4	1.1–10.1			
Private car/taxi	62.8	54.3–10.6			
Other motorised transport	30.2	23.0–38.4			
Other	3.6	0.7–16.3			
**Accompanied by a healthcare professional**					
Yes	24.9	17.8–33.6			
	**Among referred cases**		**Among non-referred facility-based births**		
	***n = 208***		***13,254***		
	**%**	**95%CI**	**%**	**95%CI**	
**Birth by caesarean section**	50.4	41.4–59.4	6.2	5.5–7.0	*p < 0.001*
**Early neonatal death**	5.4	3.1–9.3	3.8	3.3–4.4	*p = 0.217*

**Fig. 2:**
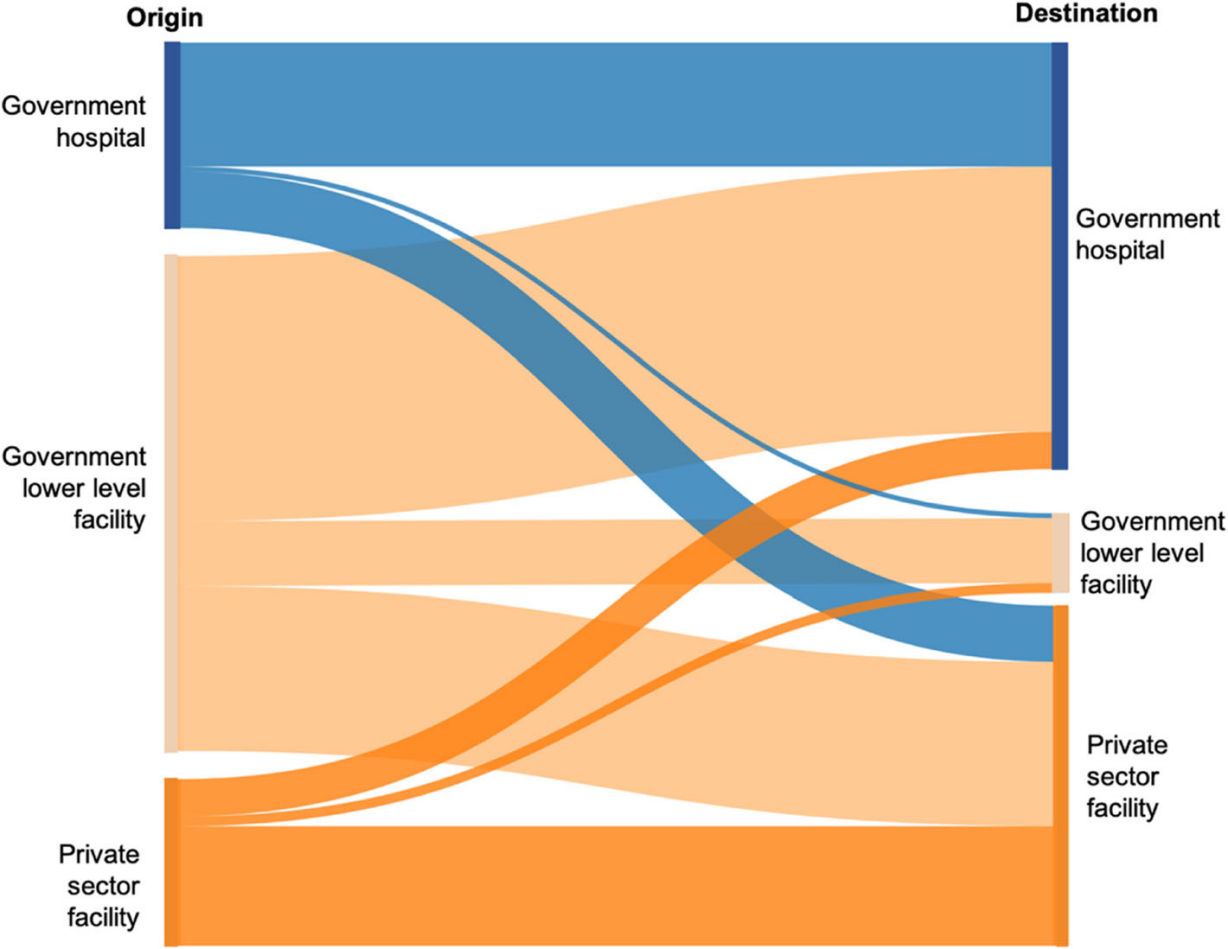
From NDHS 2018, flowchart of origin to destination facility types among women who reported being referred during labour/childbirth (*n* = 208)

Figure 2 shows the flow from the origin (referring out) to destination (receiving in/final place of birth) facility types among women referred during labour or childbirth. Most referrals from government hospitals went to other government hospitals (66.9%), but a substantial proportion of government hospital referrals gave birth in private sector facilities (30.4%). From government lower-level facilities, most referrals went to government hospitals (53.6%), but a sizeable minority went to private facilities (33.3%) and a non-negligible percentage (13.1%) went to other government lower-level facilities. The large majority (71.7%) of referrals from the private sector went to other private sector facilities; there was no distinction between level of private facility in the data.

Table 3 shows a comparison of outcomes between referred and non-referred births in health facilities. Half of referred births were delivered by caesarean, compared to 6.2% of non-referred births in health facilities (*p* < 0.001). Early neonatal mortality was slightly higher among referred facility births (54 per 1000 live births) compared to 38 per 1000 live births among non-referred facility births. However, this difference was not significant (*p* = 0.217), potentially due to the small sample size of referred cases.

### Data source 2: Lagos state CEmOC facility records – sub-national perspective

Of the 4181 pregnant women who presented with an obstetric emergency at one of the 24 government CEmOC hospitals, 1038 (25%) were referred. When compared to the reported total number of pregnant women (emergency and non-emergency) who presented at one of the 24 government CEmOC hospitals during the study period (30,129), referral rate was 3.4%. Focusing on births and excluding abortions, there were a total of 30,072 births, of which 910 (3.0%) were referred in.

Table 4 provides a summary of the demographic and pregnancy characteristics of the 1038 women in our sample who were referred. Most of the women (72.7%) were within the 20–34-year age category, 94.5% were married, 57.7% were educated and 50.7% were petty traders (self-employed in small businesses). Almost all the women (95.1%) had a singleton gestation and 52.9% delivered by caesarean.

**Table 4:** Characteristics of pregnant women presenting in emergency situations in all 24 Lagos public CEmOC facilities (August 2018–August 2019)

Background characteristics	***n =*** 1038	%	95% CI
**Reproductive age category**			
< 20 years	23	2.2	1.5–3.3
20 to 34 years	754	72.7	69.8–75.3
> 35 years	261	25.1	22.6–27.9
**Marital status**			
Married	981	94.5	92.9–95.7
Single	57	5.5	4.1–6.8
**Educational attainment**			
Primary	18	6.3	4.5–7.6
Secondary	164	57.7	54.7–59.6
Tertiary	102	36.0	34.5–38.7
Missing	754	72.6	70.1–74.3
**Employment status**			
Unemployed/Housewife	206	19.8	17.5–22.4
Student	45	4.3	3.3–5.8
Self-employed/Petty trader	526	50.7	47.6–53.7
Self-employed/Mid-High Business	92	8.9	7.3–10.8
Employed	169	16.3	14.2–18.7
**Booking status**			
Un-booked	954	92.0	90.1–93.4
Booked	84	8.0	6.6–9.9
**Parity**			
Nulliparous (0)	355	36.1	34.3–38.5
Multiparous (1–4)	604	61.4	58.7–63.8
Grand multiparous (≥5)	25	2.5	1.8–3.4
Missing	54	5.2	3.4–6.8
**Number of gestations**			
Singleton	987	95.1	93.6–96.3
Multiple (Twins/Triplets)	51	4.9	3.8–6.4
**Mode of delivery**			
Spontaneous vaginal delivery	332	32.0	29.2–34.9
Assisted Vaginal Delivery	29	2.8	1.9–3.9
Caesarean delivery	549	52.9	49.8–55.9
Evacuated before viability	128	12.3	10.5–14.5

Most of the referred women (76.0%) presented during the week, as opposed to the weekend (24.0%) (Table 5). In terms of their travel to reach facilities, the women’s medical records documented the period of the day during which the women commenced their journeys to the facility in 57.5% of the cases, but almost 97.9% did not state mode of travel to reach the facility. However, among the women with data available, 202 (33.8%) travelled in the morning. Most were referred from government lower-level facilities such as primary health centres (40.9%), followed by private hospitals (22.9%), and other government hospitals (15.9%). Fifty-two women (5.0%) were referred by more than one facility before reaching the final destination facility. Some women were reported to have made a non-referral stops en route the health facility to go to places of worship (church, mosque) or to get an ultrasound done (2.5%). Information on mode of travel to facility was only reported for 22 (2.1%) women. After delivery, 17 women (1.6%) had newborns who were transferred to other hospitals for additional neonatal care, including nursing in available incubators.

**Table 5:** Characterisation of referral documented in case records of referred pregnant women presenting in emergency situations in Lagos public CEmOC facilities and outcomes

**Background characteristics**	***n*** **= 1038**	**%**	**95% CI**				
**Day of arrival at facility**							
Weekend	249	24.0	21.5–26.7				
Weekday	789	76.0	73.3–78.5				
**Period of day that journey commenced**							
Morning (3.00 am - 12 noon)	202	19.4	17.2–21.9				
Afternoon (12 noon - 4.00 pm)	149	14.4	12.4–16.6				
Evening (4.00 pm – 8.00 pm)	138	13.3	11.4–15.5				
Night (8.00 pm – 3.00 am)	108	10.4	8.7–12.4				
Missing	441	42.5	39.5–45.5				
**Mode of travel to facility**							
Not stated	1016	97.9	96.8–98.6				
Private car	12	1.2	0.7–2.0				
Taxi	5	0.5	0.2–1.2				
Bus	2	0.2	< 0.1–0.8				
Tricycle	3	0.2	0.1–0.9				
**Type of referral institution**							
Government hospital	165	15.9	13.8–18.3				
Government lower-level facility	425	40.9	37.9–4.0				
Private hospital	238	22.9	20.5–25.6				
Clinic (Public or Private)	80	7.7	6.1–9.4				
Traditional Birth Attendant	103	9.9	8.2–11.9				
Nursing/Maternity home	27	2.7	1.8–3.2				
**Number of referrals**							
Single	986	95.0	93.5–96.2				
Multiple	52	5.0	3.8–6.5				
**Non-referral stops made en route to facility**							
Missing	1012	97.5	96.3–98.2				
Yes	26	2.5	1.5–3.6				
	**Among referred cases**			**Among non-referred cases**			
	***n = 1038***	**%**	**95% CI**	***n = 3143***	**%**	**95% CI**	***p*-value**
**Birth by caesarean section**	544	52.4	51.2–53.5	1377	43.8	41.3–45.5	*p < 0.001*
**Maternal death**	42	4.0	3.5–4.7	140	4.5	3.7–4.9	*p* = 0.576
**Fresh stillbirth**	110	13.0	12.1–14.6	145	6.3	5.8–7.7	*p < 0.001*

Comparing outcomes between referred and non-referred cases, 52.4% (95%CI 51.2–53.5) of referred women delivered by caesarean compared to 43.8% (95%CI 41.3–45.5) of non-referred births in the study hospitals (*p* < 0.001). There was no statistically significant difference in maternal deaths between referred and non-referred cases. However, there was a statistically significant difference in fresh stillbirths between referred (13.0% (95%CI 12.1–14.6)) and non-referred cases (6.3% (95%CI 5.8–7.7)) (Table 5).

### Data source 3: UBTH patient case notes – apex referral hospital perspective

A total of 253 records of women meeting the inclusion criteria were extracted and included in the study. The majority of these un-booked patients came to UBTH from another health facility (*n* = 196, 77.5%). A further 17.4% (*n* = 44) came from their home or an unspecified location, and 5.1% (*n* = 13) came from non-health institutions/locations, including church groups or the home of a TBA.

Table 6 shows the demographic and pregnancy characteristics of the full sample of un-booked women and the subset of those who were referred from a health facility (*n* = 195, excluding one woman who arrived from another health facility but was not referred formally). The vast majority of women were married and residing in Benin City, and one third of women were admitted for care for their first birth. Compared to all women in the sample, those referred from health facilities had more multiple pregnancies (11.5% vs 14.4%). More than half of women were admitted pre-term (< 37 weeks gestation), with a third of women’s pregnancies recorded as extremely or very pre-term (< 32 weeks).

**Table 6:** Characteristics of all un-booked women, and of the subset of those referred from another health facility, who were admitted to UBTH maternity ward between Dec 2017 and Jul 2018

	All un-booked women in the sample (***n*** = 253)		Referred from another health facility (n = 195)	
	%	95%CI	%	95%CI
**Reproductive age category**				
< 20 years	2.0	0.8–4.7	2.1	0.8–5.4
20 to 34 years	70.8	64.8–76.1	69.2	62.4–75.3
> 35 years	27.3	22.1–33.1	28.7	22.8–35.5
**Marital status**				
Married	94.9	91.3–97.0	95.4	91.3–97.6
Unmarried	5.1	3.0–8.7	4.6	2.4–8.7
**Residence**				
Benin City	91.3	87.1–94.2	91.3	86.4–94.5
Outside of Benin City	7.1	4.5–11.0	7.2	4.3–11.8
Unknown/missing	1.6	0.6–4.2	1.5	0.5–4.7
**Parity**				
Nulliparous (0)	32.0	26.5–38.0	31.8	25.6–38.7
Multiparous (1–4)	62.9	56.7–68.6	62.1	55.0–68.6
Grand multiparous (5+)	5.1	3.0–8.7	6.2	3.5–10.6
**Number of gestations**				
Singleton	88.5	84.0–91.9	85.6	80.0–89.9
Multiple (Twins/Triplets)	11.5	8.1–16.0	14.4	10.1–20.1
**Timing and mode of delivery**				
Delivered before arrival	6.3	3.9–10.1	5.1	2.8–9.3
Vaginal delivery	42.7	36.7–48.9	42.1	35.3–49.1
Caesarean delivery	49.0	42.9–55.2	50.8	43.7–57.8
Other^a^	0.8	0.2–3.1	1.0	0.3–4.0
Unknown/missing	1.2	0.4–3.6	1.0	0.3–4.0
**Gestational age**				
Extremely preterm (< 28 weeks)	10.3	7.1–14.7	9.7	6.3–14.8
Very preterm (28–32 weeks)	21.3	16.7–26.9	26.7	20.9–33.4
Moderate to late preterm (32–37 weeks)	19.0	14.6–24.3	20.5	15.4–26.8
Term (37+ weeks)	41.9	35.9–48.1	36.4	29.9–43.4
Postpartum arrival	3.2	1.6–6.2	2.6	1.1–6.0
Unknown/missing	4.4	2.4–7.7	4.1	2.1–8.0

^a^Includes maternal deaths prior to delivery and multiples delivery where mode of delivery at UBTH differed between twins

Among women referred from another health facility (*n* = 195), more than 80% came from another hospital (Table 7). Among the components of referral, only the presence (or absence) of a referral note was documented consistently in the medical records of the vast majority of women; nearly 60% of women were referred with a note, though the form and content of these referral notes (including previous medical interventions received) varied widely; and not all medical records still contained that original note. Whether there was notification to UBTH prior to the patient’s arrival was documented in the case notes for nearly 60% of women, though this was most often to indicate the lack of notification. In only two cases (1.0%) did the patient record indicate that UBTH received a notification, via telephone call, prior to the woman’s arrival. The type of transport used during the referral and who accompanied the woman was recorded in only 1.0 and 6.7% of patient case notes, respectively.

**Table 7:** Characterisation of referral documented in UBTH patient case notes among women referred from another health facility and outcomes

	**Referred from another health facility (*n*** **= 195)**						
		%	95%CI				
**Source facility type**							
Government hospital		33.9	27.5–40.8				
Government comprehensive health centre		4.6	2.4–8.7				
Government primary health centre		5.6	3.1–9.9				
Private hospital		46.2	39.2–53.2				
Private maternity/primary care		6.7	3.9–11.2				
Private other		2.6	1.1–6.0				
Unknown/missing		0.5	0.1–3.6				
**Notification prior to arrival**							
Telephone call		1.0	0.3–4.0				
No notification		58.5	51.4–65.2				
Unknown/missing		40.5	33.8–47.6				
**Referral note present**							
Yes^a^		59.0	51.9–65.7				
No		35.4	29.0–42.4				
Unknown/missing		5.6	3.1–9.9				
**Transport to UBTH**							
Private car/taxi		1.0	0.3–4.0				
Unknown/missing		99.0	96.0–99.8				
**Accompanied by healthcare professional**							
Yes		2.6	1.1–6.0				
No, only family accompanied		4.1	2.1–8.0				
Unknown/missing		93.3	88.8–96.1				
	**Among referred cases**			**Among non-referred cases**			
	***n***	***%***	***missing (n)***	***n***	***%***	***missing (n)***	***p-value***
**Birth by caesarean section**^b^	89	56.7	1	21	46.7	1	*p* = 0.234
**Maternal death**^**c**^	8	4.1	0	3	5.3	1	*p* = 0.706
**Stillbirth**^d^	42	23.0	2	6	11.8	1	*p* = 0.080

^a^*Includes three records where a referral note was referenced in the medical case notes, but the actual referral note was missing from the patient’s file*

^b^*Denominator: fetus alive at admission to UBTH, n = 204.*

^c^*Denominator: all UBTH maternity ward admissions, n = 253.*

^d^*Denominator: admitted in labour/before delivery, n = 234.*

Table 7 shows a comparison of obstetric outcomes between women referred from another health facility and those not referred. No significant differences in the outcomes of caesarean section or maternal death were noted between those referred and not referred. Among women admitted in labour or before delivery, there were nearly twice the stillbirths among women referred compared to those not referred (23.0% vs 11.8%, *p* = 0.080).

#### Comparative analysis

Table 8 shows a comparison of the three data sources, highlighting the strengths, gaps, and outcome measurement possibilities. One important consideration was the representativeness offered by the samples captured by the different data sources, and their comparability. The two facility-based data sources (2 and 3) were from CEmOC facilities, and women ending their referral pathways at these facilities likely represent the most severe obstetric complications. The NDHS (data source 1) offered nationally representative, population-level estimates of referral prior to childbirth care for deliveries ending in a live birth.

**Table 8:**
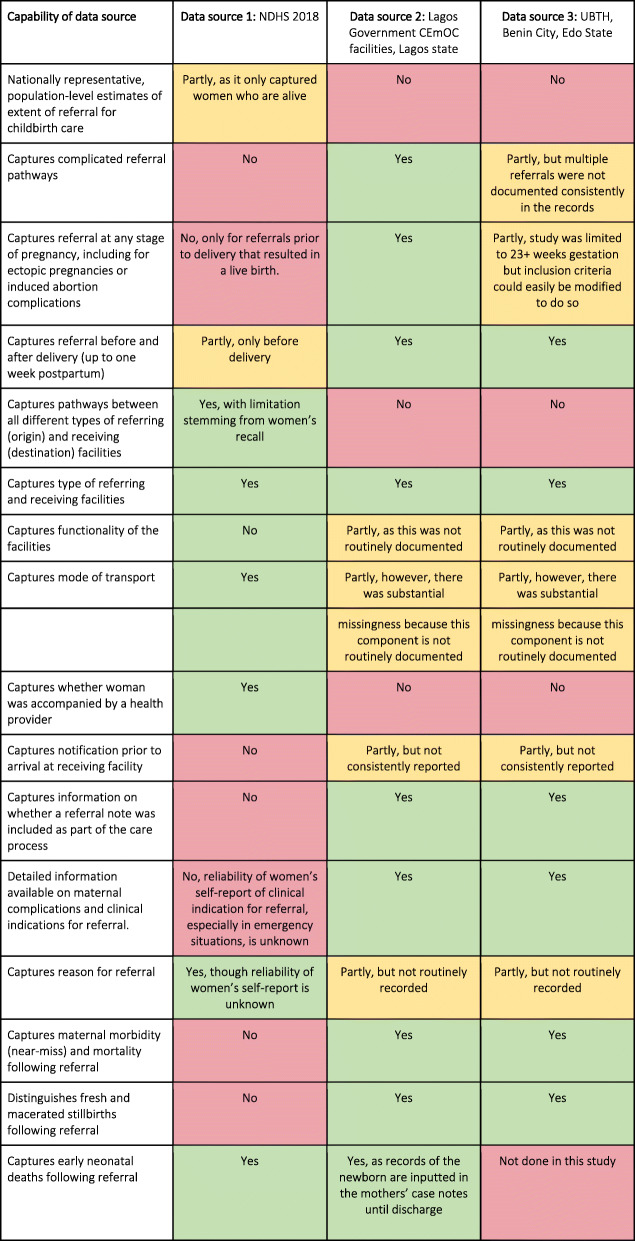
Summary of comparative strengths and gaps across the three data sources

All data sources allowed capture of direct referrals between one referring and receiving facility, while data sources 2 and 3 reported some more complicated referrals to varying degrees (Table 8). The NDHS reported an additional pattern of referral in which women bypassed government facilities (hospitals and lower-level facilities) to private sector facilities. Data source 2 showed other complicated referral pathways that included multiple referrals and neonatal referrals after delivery. It was also able to capture some informal stopovers that women made en route to the destination facilities.

Using the framework of illustrative referral pathways (Fig. 1), we examined which pathways would be captured in each of the three data sources examined here. We determined that the NDHS data source would capture the pathways of Woman A, Woman B and Woman D, as all ended in the woman’s survival and a live birth. The pathways of Woman A and Woman C would be captured in the two facility-based data sources (data sources 2 and 3), and notably, the complicated pathway of Woman C with multiple referrals could be captured as well. As the two facility-based data sources analysed were both conducted in government referral facilities, neither would capture the pathways of Woman B or Woman D, as both journeys ended in private sector facilities. Finally, Woman E’s pathway, though comparatively rare, might be captured in routine HMIS as an “out” referral, it would not be captured in any of the three data sources examined here as her pathway ended with a maternal death at home.

Facility-based data sources (in particular, data source 2) captured referral at any stage of pregnancy, including those that end as ectopic pregnancies or induced abortions. Data source 3 has the potential to capture these data, though the inclusion criteria was limited to 23+ weeks gestation for this particular study. Both facility-based data sources allowed capture of postpartum referral pathways (up to 7 days after delivery) and adverse outcomes, especially maternal morbidity and mortality, as well as stillbirths, whereas the NDHS was limited to only referrals occurring prior to delivery and only to women alive at the time of the survey whose deliveries ended in a live birth. Early neonatal deaths were captured in data source 2, as records of the newborn are inputted in the mothers’ case notes until discharge. However, the linkage between newborns’ and their mothers’ records was not possible in data source 3. The NDHS only allowed capture of early neonatal deaths following referral (Table 8).

The NDHS reported mode of transport, the reason for the referral (including problem during labour or an emergency, non-availability of a health professional, no bed space, or facility not open) and whether the woman was accompanied by a health provider, but such data were not routinely collected in facility-based data sources. On the other hand, the facility-based data sources routinely documented the clinical indication for the referral, and for the most part if a notification was sent prior to arrival and a referral note was presented on arrival (Table 8).

## Discussion

Our analysis offers a critical comparison of data sources on obstetric referral from three contemporaneous studies from a single country setting. The three data sources considered here offer different strengths and gaps, from the national to facility level, in the capture of the extent and components of obstetric referral, going back to the key referral questions that we highlighted: Who is being referred and why? Where are they coming from and going to? How are they travelling and who is travelling with them? And finally, what are the outcomes of women who are referred?

In terms of the ‘who’, we found that the DHS captured a plethora of demographic and socio-economic characteristics, allowing for a richer characterisation of those being referred. On the other hand, the facility-based data sources 2 and 3 mostly captured only basic demographic data which limits their usefulness for analysis comparing referral pathways and outcomes by sub-groups of women. When the numbers were aggregated, we found large differences in the proportion of women in each analysis who were referred, reflecting the different denominators (all facility births vs only those in CEmOC facilities, live births vs all perinatal outcomes) and different timings of the referral (only those referred prior to delivery vs those referred prior, during or up to 7 days after delivery). However, in the one instance where we were able to standardise denominators across the data sources, these (data source 1 and 2) were broadly in agreement. According to the NDHS, the proportion of live births in government hospitals which came from referrals in the South West region of Nigeria where Lagos is situated was 3.2% (95%CI 2.0–5.1). Estimates based on the Lagos State government hospital data (data source 2) was 3.4%, which is within the confidence interval estimate for the larger region. The consistency of the 2018 NDHS and the Lagos State data in measuring the proportion of women referred is particularly important and reassuring, given that these survey questions about referral for childbirth care have not yet been validated in any setting. However, we cannot rule out that women were unable to recall or report on the details of their referral accurately in the NDHS (data source 1). As to the reason (‘why’) for the referral, the facility-based data sources routinely captured clinical indications or complications and to some extent the reason for the referral, as it is part of routine clinical practice. The NDHS asked women to self-report the reason for the referral, but the reliability and understanding of this question is unknown and could be fairly inaccurate, particularly in emergencies.

To understand the potential of the various data sources to capture data on where women are referred from and referred to, we developed a conceptual visualisation of different referral pathways. Our comparative analysis showed that all three data sources allowed capture of direct referrals, from lower-level facilities to hospitals [22]. Many obstetric referral guidelines present referral as hierarchical ‘bottom-up’ travel from lower- to higher-level facilities, as stipulated in the WHO’s Practical Guide for ﻿Implementing Safe Motherhood in Countries [47]. However, our findings and others [13, 16, 28–31] show that women travel across facility levels and sectors in more complex patterns. All data sources used in our comparative study were, to a varying extent, able to reflect some complex referral pathways including lateral referrals [13]. For example, the NDHS was able to show that women bypassed government facilities (hospitals and lower-level facilities) for private sector facilities – another pattern reported in previous studies [28–31]. Data source 2 highlighted some other complicated referral pathways which included multiple referrals and neonatal referrals for reasons such as use of an incubator, which is not typically available in all hospitals [16]. It was also able to capture some informal stopovers that women made en route to the facilities. Data source 3 was able to capture the sector and level of referring facilities in more granularity and to some extent also the reasons for lateral referrals (such as hospitals lacking equipment or staff to perform caesarean sections).

Components of referral, such as transport or whether a healthcare provider accompanied the woman, were frequently missing from our two facility-based data sources, but these components were more consistently captured in women’s self-report on the NDHS. Our comparison of data sources found that facility-based records often better captured referral components that impacted clinical care, such as the presence of a referral note as such notes often included previously received interventions. During data collection at facilities, it was also observed that referral components were often recorded more completely in the medical records of women with adverse outcomes (including maternal or perinatal deaths). This potentially reflected concerns about the need to comprehensively document what had happened prior to women’s arrival at those facilities.

The data sources offered differing options for examining perinatal outcomes of women who were referred. While it was possible to report number of caesarean deliveries across all data sources, stillbirth was only reported in the facility-based data sources. Early neonatal death (within 7 days of birth) was reported in data sources 1 and 2, though later neonatal deaths (after 7 days) could also be examined in the NDHS. The facility-based data source truncation in documenting neonatal death is intuitive, in the sense that women typically would not stay in a health facility beyond one-week of delivery [48]. In the UBTH data source, the maternity ward case notes only had data on whether the newborn was born alive and left the ward alive. The newborn could have died minutes later during admission to the neonatal intensive care unit, but this was not captured in the women’s maternity ward records. Effective referral for small and sick newborns is a critical factor in reducing neonatal mortality [49], and future research should examine the possibility of linking maternity and neonatal/paediatric ward records in facility-based data sources, as well as potentially adding questions on newborn referral in household surveys. Finally, the NDHS included a survival bias, collecting data only from women who are alive at the time of the survey and only about their deliveries that ended in a live birth. Last, while we recognise that hospitals (our facility-based data sources) receive some of the most complicated obstetric emergencies, and we must highlight the unacceptably high levels of perinatal mortality among referred cases, including maternal mortality, particularly in Lagos (data 2), and neonatal mortality/stillbirth rates in both data sources 2 and 3.

As the Nigerian government invests in increasing facility-based deliveries, particularly in primary care facilities, there is a growing need to ensure safe, effective referral across all levels of the health system. Altogether, the three data sources used in our study highlight critical action points that can be useful for planning. First, between 16 and 34% of referrals in the three sources originated in government hospitals, suggesting insufficient resources even at this higher level to manage all obstetric cases. Some of the referrals between government hospitals may reflect enhanced capacity or specialisation for complex obstetric or neonatal cases at some hospitals. For example, the substantial proportion of extremely and very preterm pregnancies admitted to UBTH likely reflects the referral centre’s reputation and capacity for neonatal intensive care in Edo State. We also found large gaps in the coverage of key components of referral, finding in the 2018 NDHS analysis that only 3.4% of women used an ambulance and 24.9%, were accompanied by a healthcare professional during their referral, as well as data gaps where this information was not routinely captured in facility-based records. Additionally, the NDHS analysis revealed that a non-negligible proportion (13.1%) of women were referred between government lower-level facilities, and 5.0% of women in the Lagos State analysis had more than one referral, suggesting inefficiencies in the referral system. The health system factors behind these lateral and multiple referrals, including closed or crowded facilities, require further research to address barriers and likely delays to women receiving appropriate care, including communication between facilities during the referral process.

There are some actions that might strengthen the capabilities of the data sources for capturing referral data. With the NDHS and other future DHS surveys, there is a need to consider reframing the survey questions. Additional questions framed to trigger recall of multiple pathways and timeline of referrals, throughout the entire pregnancy and postpartum period, might help to increase capacity of the DHS to demonstrate more complicated referral pathways. However, we observed large numbers of stillbirths, including from referred cases, in the facility-based data that get missed in the restriction of maternity care and referral questions to live births. Going forward, validation studies will be needed by the DHS before recommending the new referral questions to be used more widely [50], including in cases of stillbirth. Capturing maternal deaths will remain a challenge.

For facility-based data sources, clarification of the definition of ‘booked’ v. ‘un-booked’ is important in fully understanding referral pathways and outcomes. Often being ‘booked’ means the pregnant woman is registered at a facility and has attended at least one antenatal session during which she was seen by a skilled health personnel [51–53]. However, is a woman who has been seen by a skilled health personnel at a lower-level facility in the catchment area or in hospital A and now presents in hospital B a ‘booked’ case? If yes, the medical record systems in many LMICs are rarely sufficiently coordinated to make such information linkages [22]. If no, then a clearer definition is needed to move the field forward.

Our analysis highlighted the importance of properly considering obstetric referral between facilities from different sectors and the role of household surveys, or potentially expanding facility-based data sources, to capture private sector referrals. Nigeria’s substantial private sector for maternity care (33.0% of facility deliveries) [44], particularly in urban centres, challenges efforts to formalise, monitor, and improve referral coordination. Facility-based data sources, including data sources 2 and 3 here and HMIS, are frequently limited to government/public facilities. Referrals ending in the private sector—40.2% of referred cases in the NDHS analysis—would not be captured in these government facility-based data sources. The large geographic variation in referrals to government hospitals observed in the NDHS may reflect a greater availability of private sector facilities in the South East region compared the three northern regions. In strengthening facility-based data sources in both public and private sectors, new variables that allow for comprehensive capture of referral pathways, the reason for referral and clinical indication for referral will be essential.

### Strength and limitations

A key strength of our critical comparative analysis is that we took advantage of three unique data sources available to offer perspectives on obstetric referral from the national to sub-national to facility-level. However, these data sources were not designed to answer all the questions posed in this analysis. We were limited in making direct comparisons between the results of the three sources, as they represented overlapping – but not exactly the same – time periods, denominators, and measurement approaches. Additionally, due to small sample sizes, we were limited in conducting sensitivity analyses that would have facilitated more direct comparisons between the facility-based data sources and the NDHS results from the South-West and South-South regions (of which Lagos State and Benin City, Edo State, respectively, are part). Our analysis would have also benefited from HMIS as a fourth complementary source of data on referrals. While we did not have access to these data, we included it in the visual framework to illustrate where outgoing and incoming referrals could be captured via HMIS. Finally, an important consideration of our analyses is that we cannot know if the right women were getting referred, including the extent of women with obstetric complications who should have been referred but were not. The data sources were also limited in assessing the appropriateness of the referral, particularly among referrals from similar level facilities that had the theoretical (if not practical) capability to manage the complication.

## Conclusion

In Nigeria, as in many sub-Saharan African countries, there is limited understanding of the pathways of reaching care within the obstetric referral system. As such, we potentially lose vital information which could be useful in programme and policy planning. The analyses in this paper highlight opportunities for further investigation, including referral patterns between facilities of the same level. Our unique examination of multiple data sources on obstetric referral revealed the strengths and gaps within each approach. The scale at which referral research is done (national, sub-national or intra-facility) will depend on available resources, such as the considerable expense and time needed to conduct a national household survey to the relatively low-cost pilot study at an apex facility, as well as the purpose of the investigation. Routine monitoring of obstetric referral, through for example HMIS, will require meaningful resourcing to incorporate new indicators to capture referral and its components as well as inclusion of private sector facilities. However, more rapid, cross-sectional investigations in selected facilities are also possible. This could supplement routine monitoring by targeting data collection from facilities identified as requiring more support or receiving the most urgent cases. Future research in other high mortality contexts should map and examine multiple existing data sources to allow the strengths of each to build a more complete picture of obstetric referral. Our visual framework can assist these efforts to ensure all pathways are captured, and that we retain the woman and her fetus/newborn at the start and centre of each journey.

## Supplementary Information

**Additional file 1: Appendix 1** Copy of Nigeria 2018 DHS questionnaire, excerpt of obstetric referral questions.

**Additional file 2: Appendix 2** Details of study design and sampling for UBTH patient records study (data source 3).

## Data Availability

The NDHS data (data source 1) that support the findings of this study are available from the Demographic and Health Surveys Program but restrictions apply to the availability of these data, which were used with permission for the current study, and so are not publicly available. Data are available, with permission, from the Demographic and Health Surveys Program website. The datasets from the Lagos State study (data source 2) and the UBTH study (data source 3) that support the findings of this study are available from Dr. Aduragbemi Banke-Thomas (data source 2) and Ms. Emma Radovich (data source 3) but restrictions apply to the availability of these data, which were used under license for the current study, and so are not publicly available. Data are available from the authors upon reasonable request and with permission from Dr. Banke-Thomas and Ms. Radovich for data sources 2 and 3, respectively.

## Abbreviations

CEmOC: Comprehensive emergency obstetric care
HMIS: Health management information systems
LMICs: Low- and middle-income countries
NDHS: Nigeria Demographic and Health Survey
TBA: Traditional birth attendant
UBTH: University of Benin Teaching Hospital
WHO: World Health Organization
