# Supplementary material for: Critical comparative analysis of data sources toward understanding referral during pregnancy and childbirth: three perspectives from Nigeria
Source: BMC Health Serv Res. 2021 Sep 6;21:927. doi: 10.1186/s12913-021-06945-9 (PMC8420846; doi:10.1186/s12913-021-06945-9)
Supplement: Supplementary file 1 — Additional file 1: Appendix 1 Copy of Nigeria 2018 DHS questionnaire, excerpt of obstetric referral questions. [file 12913_2021_6945_MOESM1_ESM.pdf]

TITLE:

Critical comparative analysis of data sources toward understanding referral during pregnancy and childbirth: three perspectives from Nigeria

AUTHORS: Emma **Radovich**[1]*/Aduragbemi **Banke-Thomas**[2]*, Oona M.R. **Campbell**[1], Michael **Ezeanochie**[3], Uchenna **Gwacham-Anisiobi**[4], Adedapo B.A. **Ande**[3], Lenka **Benova**[1,5]

*joint first

[1] Faculty of Epidemiology and Population Health, London School of Hygiene and Tropical Medicine London, United Kingdom

[2] LSE Health, London School of Economics and Political Science, London, United Kingdom

[3] Department of Obstetrics and Gynaecology, University of Benin Teaching Hospital, Benin, Edo State, Nigeria

[4] Health Strategy and Delivery Foundation, Owerri, Imo State, Nigeria

[5] Department of Public Health, Institute of Tropical Medicine, Antwerpen, Belgium

APPENDIX 1 Copy of Nigeria 2018 DHS questionnaire, excerpt of obstetric referral questions

SECTION 4. PREGNANCY AND POSTNATAL CARE

NO.	QUESTIONS AND FILTERS	LAST BIRTH<br>NAME ______	NEXT-TO-LAST BIRTH<br>NAME ______
430	Where did you give birth to (NAME)?<br><br>PROBE TO IDENTIFY THE TYPE OF SOURCE.<br><br>IF UNABLE TO DETERMINE IF PUBLIC OR PRIVATE SECTOR, WRITE THE NAME OF THE PLACE.<br><br>______<br>(NAME OF PLACE)	**HOME**<br>HER HOME . . . . 11<br>OTHER HOME . . . . 12<br>(SKIP TO 434)<br><br>**PUBLIC SECTOR**<br>GOVERNMENT HOSPITAL . . 21<br>GOVERNMENT HEALTH CENTER . . . . 22<br>GOVERNMENT HEALTH POST . . . . 23<br>OTHER PUBLIC SECTOR ______ 26<br>(SPECIFY)<br><br>**PRIVATE MEDICAL SECTOR**<br>PRIVATE HOSPITAL/ CLINIC . . . . 31<br>OTHER PRIVATE MEDICAL SECTOR ______ 36<br>(SPECIFY)<br><br>OTHER ______ 96<br>(SPECIFY)<br>(SKIP TO 434)	**HOME**<br>HER HOME . . . . 11<br>OTHER HOME . . . . 12<br>(SKIP TO 459)<br><br>**PUBLIC SECTOR**<br>GOVERNMENT HOSPITAL . . 21<br>GOVERNMENT HEALTH CENTER . . . . 22<br>GOVERNMENT HEALTH POST . . . . 23<br>OTHER PUBLIC SECTOR ______ 26<br>(SPECIFY)<br><br>**PRIVATE MEDICAL SECTOR**<br>PRIVATE HOSPITAL/ CLINIC . . . . 31<br>OTHER PRIVATE MEDICAL SECTOR ______ 36<br>(SPECIFY)<br><br>OTHER ______ 96<br>(SPECIFY)<br>(SKIP TO 459)
430A	Did you move from another health facility to come to this facility or did you go directly from home to this facility, or from somewhere else that was not a health facility?	CAME FROM ANOTHER HEALTH FACILITY . . . . 1<br>CAME FROM HOME . . . . 2<br>CAME FROM OTHER NON-FACILITY LOCATION . . 3<br><br>DON'T KNOW . . . . 8<br>(SKIP TO 430F)	CAME FROM ANOTHER HEALTH FACILITY . . . . 1<br>CAME FROM HOME . . . . 2<br>CAME FROM OTHER NON-FACILITY LOCATION . . 3<br><br>DON'T KNOW . . . . 8<br>(SKIP TO 430F)
430B	Which health facility referred or send you to this facility where you gave birth to (NAME)?<br><br>PROBE TO IDENTIFY THE TYPE OF SOURCE.<br><br>IF UNABLE TO DETERMINE IF PUBLIC OR PRIVATE SECTOR, WRITE THE NAME OF THE PLACE.<br><br>______<br>(NAME OF PLACE)	**PUBLIC SECTOR**<br>GOVERNMENT HOSPITAL . . 21<br>GOVERNMENT HEALTH CENTER . . . . 22<br>GOVERNMENT HEALTH POST . . . . 23<br>OTHER PUBLIC SECTOR ______ 26<br>(SPECIFY)<br><br>**PRIVATE MEDICAL SECTOR**<br>PRIVATE HOSPITAL/ CLINIC . . . . 31<br>OTHER PRIVATE MEDICAL SECTOR ______ 36<br>(SPECIFY)<br><br>NO FORMAL REFERRAL . . . . . 41<br><br>OTHER ______ 96<br>(SPECIFY)	**PUBLIC SECTOR**<br>GOVERNMENT HOSPITAL . . 21<br>GOVERNMENT HEALTH CENTER . . . . 22<br>GOVERNMENT HEALTH POST . . . . 23<br>OTHER PUBLIC SECTOR ______ 26<br>(SPECIFY)<br><br>**PRIVATE MEDICAL SECTOR**<br>PRIVATE HOSPITAL/ CLINIC . . . . 31<br>OTHER PRIVATE MEDICAL SECTOR ______ 36<br>(SPECIFY)<br><br>NO FORMAL REFERRAL . . . . . 41<br><br>OTHER ______ 96<br>(SPECIFY)
430C	Why did you move from this facility to the facility where you gave birth to (NAME)?	PROBLEM DURING LABOR/ EMERGENCY . . . . 1<br>HEALTH PROFESSIONAL NOT AVAILABLE . . . . 2<br>FACILITY TOO CROWDED/ NO BED AVAILABLE . . . . . 3<br>FACILITY NOT OPEN . . . . 4<br><br>OTHER ______ 6<br>(SPECIFY)	PROBLEM DURING LABOR/ EMERGENCY . . . . 1<br>HEALTH PROFESSIONAL NOT AVAILABLE . . . . 2<br>FACILITY TOO CROWDED/ NO BED AVAILABLE . . . . . 3<br>FACILITY NOT OPEN . . . . 4<br><br>OTHER ______ 6<br>(SPECIFY)
430D	Did a health worker go with you when you moved to the facility where you gave birth to (NAME)?	YES . . . . 1<br>NO . . . . 2<br>DON'T KNOW 8	YES . . . . 1<br>NO . . . . 2<br>DON'T KNOW 8

APPENDIX 1 Copy of Nigeria 2018 DHS questionnaire, excerpt of obstetric referral questions

430E	What means of transportation did you use to get from the facility that referred you to the faclity where you gave birth to (NAME)?	**MOTORISED**<br>AMBULANCE . . . . A<br>PRIVATE CAR/TRUCK . . . . . B<br>TAXI/PAID DRIVER . . . . C<br>TRICYCLE . . . . D<br>MOTORCYCLE/SCOOTER . . E<br>BOAT WITH MOTOR . . . . . F<br>PUBLIC TRANSPORT/BUS . . G	**MOTORISED**<br>AMBULANCE . . . . A<br>PRIVATE CAR/TRUCK . . . . . B<br>TAXI/PAID DRIVER . . . . C<br>TRICYCLE . . . . D<br>MOTORCYCLE/SCOOTER . . E<br>BOAT WITH MOTOR . . . . . F<br>PUBLIC TRANSPORT/BUS . . G
430F	What means of transportation did you use to get to the health facility where you gave birth to (NAME)?<br><br>PROBE FOR THE TYPE (S) OF TRANSPORT USED AND RECORD ALL MENTIONED.	**NOT MOTORISED**<br>BICYCLE . . . . H<br>CANOE/BOAT WITHOUT MOTOR . . . . I<br>ANIMAL-DRAWN CART . . . . . J<br>WALKING (ON FOOT) . . . . . K<br>CARRIED . . . . L<br><br>OTHER ________________ X<br>(SPECIFY)<br>DON'T KNOW . . . . Z	**NOT MOTORISED**<br>BICYCLE . . . . H<br>CANOE/BOAT WITHOUT MOTOR . . . . I<br>ANIMAL-DRAWN CART . . . . . J<br>WALKING (ON FOOT) . . . . . K<br>CARRIED . . . . L<br><br>OTHER ________________ X<br>(SPECIFY)<br>DON'T KNOW . . . . Z
430G	How long did it take for you to decide to go and reach the health facility?<br><br>IF LESS THAN ONE HOUR, RECORD IN MINUTES.	MINUTES . . . . 1 [ ][ ]<br>HOURS . . . . 2 [ ][ ]<br><br>DON'T KNOW 998	MINUTES . . . . 1 [ ][ ]<br>HOURS . . . . 2 [ ][ ]<br><br>DON'T KNOW 998

Reference:

National Population Commission, ICF International. Nigeria Demographic and Health Survey 2018. Abuja, Nigeria and Rockville, Maryland, USA; 2019. Excerpt from Appendix E, pages 603-605. Full report available from: https://dhsprogram.com/pubs/pdf/FR359/FR359.pdf
