# Supplementary material for: Critical comparative analysis of data sources toward understanding referral during pregnancy and childbirth: three perspectives from Nigeria
Source: BMC Health Serv Res. 2021 Sep 6;21:927. doi: 10.1186/s12913-021-06945-9 (PMC8420846; doi:10.1186/s12913-021-06945-9)
Supplement: Supplementary file 2 — Additional file 2: Appendix 2 Details of study design and sampling for UBTH patient records study (data source 3). [file 12913_2021_6945_MOESM2_ESM.pdf]

TITLE:

Critical comparative analysis of data sources toward understanding referral during pregnancy and childbirth: three perspectives from Nigeria

AUTHORS: Emma **Radovich**[1]*/Aduragbemi **Banke-Thomas**[2]*, Oona M.R. **Campbell**[1], Michael **Ezeanochie**[3], Uchenna **Gwacham-Anisiobi**[4], Adedapo B.A. **Ande**[3], Lenka **Benova**[1,5]

*joint first

[1] Faculty of Epidemiology and Population Health, London School of Hygiene and Tropical Medicine London, United Kingdom

[2] LSE Health, London School of Economics and Political Science, London, United Kingdom

[3] Department of Obstetrics and Gynaecology, University of Benin Teaching Hospital, Benin, Edo State, Nigeria

[4] Health Strategy and Delivery Foundation, Owerri, Imo State, Nigeria

[5] Department of Public Health, Institute of Tropical Medicine, Antwerpen, Belgium

APPENDIX 2 Details of study design and sampling for UBTH patient records study (data source 3)

University of Benin Teaching Hospital (UBTH) patient case notes

Setting & study design

UBTH is the top referral centre for maternity services in Edo State. Based on unpublished data, approximately one quarter of UBTH maternity care patients are referred cases for urgent intervention, and more than 60% of these referrals are from health facilities within the UBTH catchment area. We conducted a retrospective review of the medical records of 'unbooked' women (that is, they did not receive antenatal care at UBTH) who were admitted between 23+ weeks gestation to up to seven days following delivery to the UBTH maternity ward between 1 December 2017 and 31 July 2018. We employed a census approach of extracting all records identified and meeting the inclusion criteria.

The UBTH Department of Obstetrics and Gynaecology monthly register book for women admitted to the UBTH maternity ward was used to identify the case numbers of unbooked patients, whose medical case files were requested from the hospital records archive. Medical records of women meeting the inclusion criteria were reviewed by UBTH junior doctor research assistants, and demographic, clinical management, referral, obstetric complication and maternal and perinatal outcome data were extracted from the largely free-form notes. Where needed to clarify notes in the case record or accompanying documents, research assistants spoke to the relevant provider or supervisor of care.

Sample size

Two major challenges hindered reaching the desired sample size of 340 patient records eligible for inclusion. The first was that UBTH admitted no eligible patients during May 2018 as a result of a national strike by healthcare staff at federal facilities, which included UBTH. Secondly, there were substantial challenges in locating patient records in the UBTH archives. Errors were noted in the monthly register books used to identify records for inclusion, including some patients recorded as admitted to the maternity ward but where a case record number was not recorded in the register. Due to concerns about bias in records that we were unable to find in the archives, only months where greater than 70% of requested records could be located were included in the study, resulting in 253 patient records:

Month of admission to UBTH	Records requested from archives	Requested records accounted for[1]	Requested records missing (% of requested)	Non-requested records meeting inclusion criteria[2]	Total records extracted	TOTAL records included in study
Jul-18	52†	37	15 (29%)	9	38	38
Jun-18	31	22	9 (29%)	15	39	38
May-18	0	-	-	-	-	-
Apr-18	23	20	3 (13%)	15	31	31
Mar-18	45‡	32	13 (29%)	4	33	31
Feb-18	48	43	5 (10%)	5	49	48
Jan-18	37	27	10 (27%)	5	32	31
Dec-17	43	35	8 (19%)	2	36	36
TOTAL	279	216	63 (23%)	55	258	253

[1]Records were located in the archives and either met the inclusion criteria and were extracted or were excluded.

[2]These records were found in the archives, met the inclusion criteria and were extracted, but the patient record number does not appear on the originally requested list.

†One requested record was missing patient record number.

‡Two requested records were missing patient record number.

APPENDIX 2 Details of study design and sampling for UBTH patient records study (data source 3)

Definitions and missing data

Women were classified as coming from another health facility, a non-facility location (such as church or home of a traditional birth attendant) or from home. Among women arriving from another health facility (n=196), all but one (who left the health facility due to dissatisfaction with care) were classified as having been referred from the health facility. Among women referred from another health facility, the name or description of the facility from the case record was extracted as written. The facilities were categorised into six facility types based on the investigators' knowledge of public and private sector health facilities in Edo State: government hospital, government comprehensive health centre (which offer some surgeries including csections), government primary health centre, private hospital, private maternity/primary care, and private other (comprising private sector facilities where the facility name or level could not be determined). Less than 1% of health facility referrals were missing facility name or unable to be classified.

We grouped pregnancies by estimated gestational age of the pregnancy on admission using WHO definitions for pre-term birth: extremely preterm (<28 weeks), very preterm (28 to 32 weeks), moderate to late preterm (32 to 37 weeks), and term (37 weeks+) (27). We grouped women admitted postpartum into a separate category as 8 of 16 cases were missing gestational age. Women's age, marital status, residence and parity were based on self-report in the patient history case notes. One woman in the sample was recorded as having had triplets; to protect her privacy, this was recoded to a combined category for multiple pregnancy (twins and triplets).

For type of delivery, we classified births as vaginal (combining spontaneous and instrumental vaginal deliveries), c-section, other and those delivering prior to admission at UBTH. 1% of women were missing or had an unknown type of delivery, and this included two instances of women discharged (against medical advice) prior to delivery. The category of 'other' type of delivery included cases of differing modes of delivery between multiple births and one maternal death prior to delivery. Whether women delivered prior to arrival at UBTH was recorded inconsistently in the dataset. Women were re-classified as having delivered prior to arrival at UBTH – even if the mode of delivery was noted – if post-partum complications were documented on arrival, such as post-partum haemorrhage, retained placenta or baby was stillborn prior to admission (n=9). Among women arriving to UBTH in labour or prior to delivery, 1.3% (n=3) records were missing whether the baby was born alive, including the two instances of women discharged prior to delivery. Among all admissions, one record was missing whether the woman was alive at discharge from UBTH as pages were missing from the case notes.

No records were missing booking status or date of admission; 11 records (4.4%) were missing gestational age and were retained in the analysis. The extent of missingness was described for all variables.
